# L-Histidine Inhibits Biofilm Formation and *FLO11*-Associated Phenotypes in *Saccharomyces cerevisiae* Flor Yeasts

**DOI:** 10.1371/journal.pone.0112141

**Published:** 2014-11-04

**Authors:** Marc Bou Zeidan, Giacomo Zara, Carlo Viti, Francesca Decorosi, Ilaria Mannazzu, Marilena Budroni, Luciana Giovannetti, Severino Zara

**Affiliations:** 1 Dipartimento di Agraria, University of Sassari, Sassari, Italy; 2 Dipartimento di Scienze delle Produzioni Agroalimentari e dell'Ambiente, University of Florence, Firenze, Italy; CNR, Italy

## Abstract

Flor yeasts of *Saccharomyces cerevisiae* have an innate diversity of *FLO11* which codes for a highly hydrophobic and anionic cell-wall glycoprotein with a fundamental role in biofilm formation. In this study, 380 nitrogen compounds were administered to three *S. cerevisiae* flor strains handling *FLO11* alleles with different expression levels. *S. cerevisiae* strain S288c was used as the reference strain as it cannot produce FLO11p. The flor strains generally metabolized amino acids and dipeptides as the sole nitrogen source, although with some exceptions regarding L-histidine and histidine containing dipeptides. L-histidine completely inhibited growth and its effect on viability was inversely related to *FLO11* expression. Accordingly, L-histidine did not affect the viability of the Δ*flo11* and S288c strains. Also, L-histidine dramatically decreased air–liquid biofilm formation and adhesion to polystyrene of the flor yeasts with no effect on the transcription level of the *FLO11* gene. Moreover, L-histidine modified the chitin and glycans content on the cell-wall of flor yeasts. These findings reveal a novel biological activity of L-histidine in controlling the multicellular behavior of yeasts.

## Introduction

Nitrogen starvation triggers cell adhesion and multicellular growth in different yeast species [1]–[2]. In *Saccharomyces cerevisiae* flor strains, nitrogen limitation induces activation of the *FLO11* gene and formation of the air–liquid biofilm, or flor velum [3]. The General Amino Acids Control (GAAC) pathway and/or the plasma membrane localized Ssy1-Ptr3-Ssy5 (SPS) sensor, responsible for nitrogen sensing, are also involved in the regulation of *FLO11* gene expression [4]–[7]. The *FLO11* gene codes for an extensively O-mannosylated cell-wall protein that triggers cell–cell and cell–surface adhesion and air–liquid biofilm formation in flor yeast strains [8]–[9]. The phosphorylation of the mannosyl side chains on the outer surface of yeast creates abundant negatively charged groups and provides yeast with an anionic surface charge at pH≥3 [10]–[11]. Therefore, nonspecific interactions, such as hydrophobic and electrostatic interactions, are also involved in cellular adhesion and binding [12]–[14]. Indeed, *flo11* mutants show a drastic decrease in cell-wall O-mannosylation sites, loss of adhesion and biofilm formation ability, and loss of affinity for hydrophobic solvents [8], [15]–[20]. These phenotypes also appear to be greatly influenced by gene length and expression levels of the *FLO11* gene [21]. Along with the *FLO11* gene response to adverse environments, cell-wall components such as chitin, β-glucan, and mannosyl residues are also involved in the process of adaptation to environmental stress, which is orchestrated mainly by the cell-wall integrity pathway [22]–[23].

In the present study, the effects of 380 nitrogen sources were evaluated for flor strains with different *FLO11* alleles, using Phenotype Microarray (PM) technique. The data show high variability in nitrogen metabolism among the tested strains. The flor strains metabolized a wide range of nitrogen sources, but remarkably, did not metabolize dipeptides containing L-histidine. Interestingly, subsequent biofilm formation and adhesion to polystyrene analysis explored a novel role of L-histidine in reducing dramatically these *FLO11*-related phenotypes.

## Materials and Methods

### Yeast strains

The yeast strains used in this study are reported in Table 1. The A9, M23 and V80 strains are flor strains that were isolated from different Vernaccia wineries in Sardinia, and that differ in their *FLO11* gene lengths (5, 3.1 and 6 kb) and expression levels (19.04, 7.2, 0.25 AU) [21]. Strain 3238-32 is a haploid derivative of A9, and strain 3238-32Δ*flo11* is a derivative of 3238-32 that was obtained by Zara *et al.* (2005) [16] and that lacks functional Flo11p. The S288c strain has a mutation in the *FLO8* gene that disables *FLO11* expression [24].

**Table 1:** *Saccharomyces cerevisiae* strains used in this study.

Strain	Genetic background	Reference
A9	Wild flor strain of *S. cerevisiae* isolated from Arvisonadu wine	Zara et al., 2009
M23	Wild flor strain of *S. cerevisiae* isolated from Malvasia wine	Zara et al., 2009
V80	Wild flor strain of *S. cerevisiae* isolated from Vernaccia wine	Zara et al., 2009
3238-32	*MATα leu2-*Δ*1 lys2-801 ura3-52*	Zara et al., 2005
3238-32Δ*flo11*	*MATα leu2-*Δ*1 lys2-801 flo11*Δ*::URA3 ura3-52*	Zara et al., 2005
S288c	*MATα SUC2 gal2 mal mel flo1 flo8-1 hap1 ho bio1 bio6*	Mortimer & Johnston, 1986

### Media and culture preparation

The media used in this study were YPD medium (1% yeast extract, 2% peptone, 2% glucose), 20% YPD medium (0.2% yeast extract, 0.4% peptone, 0.4% glucose), Biolog specific IFY-0 with the appropriate additives (1× IFY-0 culture medium, 20 mM D-glucose, 5 mM KH_2_PO_4_, 2 mM NaSO_4_ and 1× DyeD Biolog), buffered SC minimal medium (0.17% yeast nitrogen base [YNB] without ammonium sulfate and amino acids, 0.5% ammonium sulfate, 20 mM glucose, and aliquots of 0.1 M C_6_H_8_O_7_.H_2_O and 0.2 M Na_2_HPO_4_ stock solutions that were added to buffer the medium at pH 3, 4, 5 and 6, based on instructions from the Sigma-Aldrich buffer reference center) and flor medium (0.17% YNB without ammonium sulfate and amino acids, 0.5% ammonium sulfate, 4% ethanol) [16]. The supplemented amino acids were added at standard concentrations, as required. Unless otherwise stated, the cell cultures were prepared by overnight incubation in 5 mL YPD at 30°C and with 200 rpm agitation, and then aliquots of the cultures were inoculated in fresh YPD medium for 4 h under the same conditions, to reach the exponential phase (optical density at 600 nm [OD_600_], 0.4 to 0.5). The cell cultures were than washed twice with sterile water, the OD_600_ was measured, and the appropriate cell concentrations were inoculated into the different media.

### Phenotype microarray

The phenotype microarray (PM) was carried out on microtiter plates (PM3B, PM6, PM7 and PM8) purchased from Biolog, Omnilog (Hayward, CA, USA), which allowed the screening of 380 different nitrogen sources, including single amino acids, di/tripeptides, purines, pyrimidines and monoamines [25]. The PM technology measures active metabolism by recording the irreversible reduction of tetrazolium violet to formazan, as indirect evidence of respiratory activity. The strains were grown on YPD agar plates overnight at 30°C and resuspended in 15 mL nutrient supplement solution (9.12 mM L-leucine, 5.76 mM L-lysine, 2.59 mM uracil) using a sterile cotton swab, and the cell density was adjusted to 62% transmittance on a Biolog turbidimeter, as equivalent to OD_600_ 0.22 (2–3×10^6^ cells/mL). The final inoculating fluids were prepared by diluting the cell suspension 48-fold (62% transmittance in nutrient supplement solution) in IFY-0 apposite culture medium. Then 100 uL of the final inoculating fluids were seeded into the Biolog PM3B, PM6, PM7 and PM8 plates. Next, the PM plates were sealed with Breath-easy gas permeable membrane (Sigma-Aldrich, Milan, Italy), and incubated statically at 30°C in an Omnilog Reader for 96 h. Each experiment was performed in duplicate. The quantitative color changes were recorded automatically every 15 min using a CCD camera, to generate a growth curve for each well. The metabolism of the control wells was considered as the zero point for the other wells. The kinetic responses of the strains in each well were analyzed using the Omnilog-PM software (Biolog, Inc., Hayward, CA, USA).

For the analysis with the nitrogen metabolic assays, two kinetic parameters were used: S, the slope of the kinetic curve; and ΔH, the difference between the maximum and the minimum heights of the kinetic curve. Both of these parameters were combined to calculate the nitrogen activity index, I_N_, defined as in Equation (1):
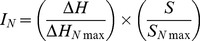
(1)where ΔH_Nmax_ and S_Nmax_ are the highest ΔH and the highest slope, respectively, recorded in the nitrogen panels (PM3B, PM6, PM7 and PM8). The I_N_ ranged between 0 (no metabolic activity) and 1 (maximum metabolic activity), and was used as a parameter for the cluster analysis of the metabolic profiles of the strains grown on the nitrogen sources. As the negative controls (wells A01 in PM3, PM6, PM7, PM8, without a nitrogen source) showed high background; the nitrogen sources were considered to be used when I_N_ was >0.33. Cluster analysis was performed using the Bionumeric software (Applied Maths, Inc, Austin, TX, USA), using Pearson's coefficient and the Unweighted Pair Group Method with Arithmetic Mean (UPGMA). The cophenetic correlation coefficient was computed to evaluate the quality of the cluster analysis.

### Antimicrobial activity of L-histidine and L-histidine–containing dipeptides

Dose response assays were carried out in 96-well microtiter plates. Aliquots of 135 µL of the cell suspensions containing 10^4^ cells/mL in 20% YPD were mixed in the microtiter plate wells with 15 µL 10× concentrated L-histidine from serial two-fold dilutions. Distilled water was used instead of L-histidine in the control wells. All of the samples were prepared in triplicate. The same test was repeated with the dipeptides histidine–methionine (HM), histidine–valine (HV) and histidine–serine (HS) at ≥95% purity (GenScript, NJ, USA), chosen for being representative of the L-histidine containing dipeptides tested by the PM analysis, and for their different physico-chemical features. The microtiter plates were incubated statically at 30°C for 48 h. Growth was measured automatically every 30 min at OD_600_ using a SPECTROstar nano microplate spectrophotometer (BMG Labtech, Ortenberg, Germany). The average of specific growth rates and the lag time of the curves obtained were analyzed using the DMFit software [26].

### Biofilm formation, adhesion ability, and cell viability in the presence of L-histidine and L-histidine–contained dipeptides

Biofilm formation was analyzed in 24-well microtiter plates in the presence of L-histidine, HM, HV and HS, as follows. Cell suspensions containing 5×10^6^ cells/mL were prepared in flor medium, and aliquots of 1350 µL were mixed in 24-well microtiter plates with 150 µL 10× concentrated L-histidine or dipeptides stock, to a final concentration of 10 mM; distilled water was added to the control wells. The plates were prepared in duplicate and were incubated statically at 30°C for 5 days. The biofilm weights were measured and calculated as described by Zara et al. (2010) [27], and the cell viability was determined by serial dilution spot tests on YPD agar plates.

The yeast adherence to polystyrene was evaluated as described by Reynolds and Fink (2001) [15], with some modifications. Briefly, cell cultures were prepared as for the biofilm formation test, and 90 µL cell suspensions containing 5×10^6^ cells/mL in flor medium were placed into the 96-well polystyrene microtiter plates with 10 µL 10× concentrated L-histidine, HM, HV and HS solutions, to a final concentration of 10 mM. The cell suspensions were incubated statically at 30°C for 48 h. An equal volume of 1% (w/v) crystal violet was added to each well. After 30 min, the wells were washed with sterile water, and the adherence of cells was quantified by solubilizing the retained crystal violet in 100 mL 10% (w/v) SDS and an equal volume of sterile water. After 30 min, 50 µL of these solutions were transferred to fresh 96-well polystyrene microtiter plates, and then A_570_ and A_590_ were measured spectrophotometrically.

### Quantitative real-time PCR

The yeast strain A9 was grown overnight and refreshed as described above. Aliquots of 2.7 mL flor medium containing 5×10^6^ cells/mL were mixed with 300 µL of sterile water (Ctrl) or with a 10× L-histidine (final concentration 10 mM), and further incubated for 48 hours at 30°C without agitation. Three independent biological replicates were conducted for each sample. Cells were collected by centrifugation and kept at −80°C until processed for RNA isolation. Total RNA was extracted using the Aurum Total RNA Mini Kit (Bio-Rad, Milan, Italy). Two micrograms of total RNA were retro transcribed with iscript cDNA synthesis kit (Invitrogen Life Technologies, Milan, Italy). Quantitative real time PCR (qPCR) was performed using a CFX Connect Real-Time PCR System (Bio-Rad, Milan, Italy), according to manufacturer's protocols using the Syber GreenER qPCR SuperMix for iCycler (Invitrogen Life Technologies, Milan, Italy), with the following thermal profile: activation step (95°C for 10 min); amplification step (40 cycles of 95°C for 10 s, 56°C for 10 s, 72°C for 10 s); melting curve program (95°C for 10 s, 60°C for 15 s, 95°C with a heating rate of 0.1°C/s); and cooling step (40°C for 30 s). Primers for the target gene *FLO11*, as well as *ALG9*, *TAF10* and *UBC6* as independent reference genes [28]–[29], were designed to an equal annealing temperature of 56°C (Table S1). The quantification cycle point (Cq) for each transcript was obtained using the Bio-Rad CFX Manager software (Bio-Rad, Milan, Italy). Three technical repeats of each one of the three biological replicates were conducted. Normalization of the expression levels among different samples was carried out by considering the geometric mean of the expression levels of the three reference genes *ALG9*, *TAF10* and *UBC6*. *FLO11* relative expression levels were determined using the formula proposed by Pfaffl et al. (2001) [30].

### Flow cytometry analysis of mannose residues

Flow cytometry techniques were used to quantify the mannose residues of the cells in the presence of L-histidine. Cell suspensions of 5×10^6^ cells/mL in flor medium were incubated for 3 h without or with 10 mM L-histidine, washed, and resuspended in phosphate-buffered saline, pH 7.2 (1.18 g/L of Na_2_HPO_4_-2H_2_O, 0.22 g/L NaH_2_PO_4_, 8.5 g/L NaCl). Then 10 µL concanavalin A lectin labeled with fluorescein isothiocyanate (ConA-FITC; FITC contents 3.6 mol/mol lectin; Sigma-Aldrich Milan, Italy; stock solution, 1 mg conjugate/mL) was added, and the cells were incubated for 20 min at room temperature, in the dark. After this incubation, the samples were immediately analyzed, using a BD FACSCalibur flow cytometer (BD Biosciences, San Jose, USA). The acquisition protocol of 20,000 cells/sample was defined at FL1-h after measuring the background fluorescence and the maximum fluorescence of each strain, to standardize the fluorescence activity between them. The data were analyzed using the Expo32 software included with the cytometer.

### Fluorescence microscopy

Fluorescence microscopy was used to quantify the chitin residues of all of the tested yeast strains in the presence of L-histidine. One mL flor medium containing 5×10^6^ cells/mL was incubated without or with 10 mM L-histidine for 3 h at 30°C. In separate experiments, strains 3238-32 and 3238-32Δ*flo11* were incubated in SC minimal medium buffered to pH 3 and 6 with 1 mM tetramethylrhodamine-labeled histidine–histidine dipeptide (TMR-HH; ≥95% purity; GenScript, NJ, USA) for 3 h at 30°C. After the incubations, aliquots of 25 µM calcofluor white (CFW) were added for 5 min. The cells were washed and examined using a YM10 monochrome fluorescence CCD camera (BX61 motorized system microscope, Olympus, Tokyo, Japan) with excitation/emission wavelengths of 395/440 nm for CFW detection, and 550/573 nm for TMR detection. Differential interference contrast and fluorescence images were captured under the 100× objective using the imaging software Cell* for life science microscopy (Olympus, Tokyo, Japan). The captured photographs were merged using MacBiophotonics MBF ImageJ software.

### Cell surface charge variation of 3238-32 and the flo11 mutant in minimal medium at different pHs

The growth and the cell surface net charge of strains 3238-32 and its isogenic 3238Δ*flo11* in minimal medium plus 5 mM L-histidine were measured at different pHs. The cells (10^4^ cells/mL) were incubated in a series of buffered SC minimal media plus 5 mM L-histidine. The cells were grown in 96 wells microtiter plates, statically at 30°C for 48 h. Their growth was monitored by measuring the OD_600_ in a SPECTROstar nano microplate spectrophotometer (BMG Labtech, Ortenberg, Germany). Replicates of each experiment were used to measure the cell surface net charge Z-potential using a Zetasizer Nano (Zetasizer Ver. 6.20 Malvern Instruments, Malvern, UK), after 48 h of incubation. All of the measurements represent means and standard deviations of three replicates.

## Results

### Flor strains differ significantly in catabolism of nitrogen sources

**Figure 1:**
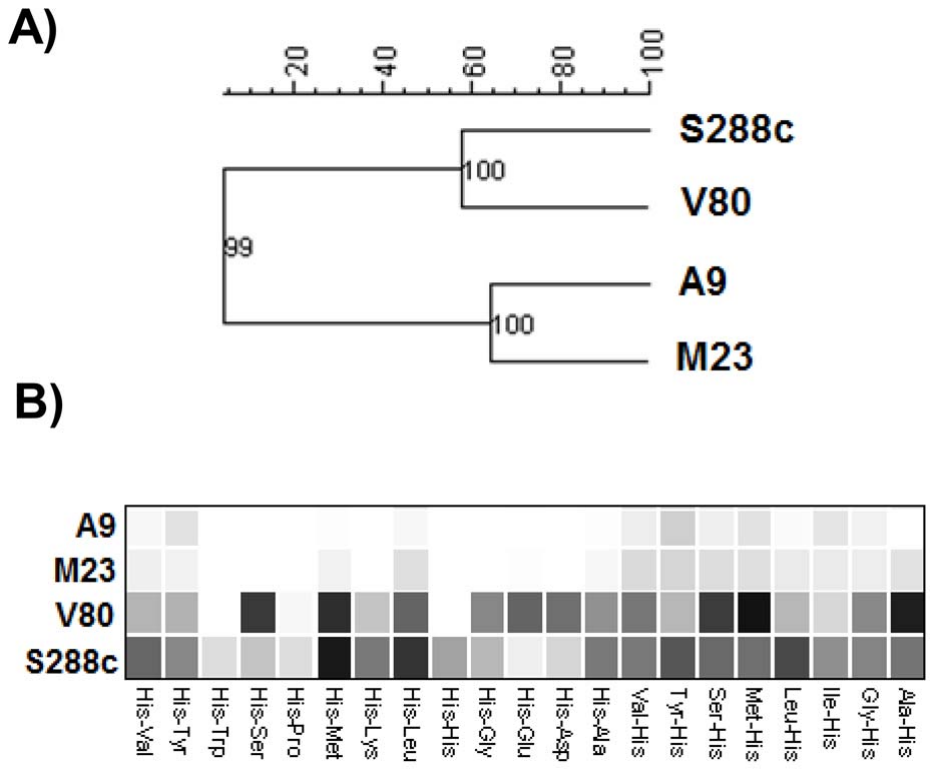
High throughput and cluster analysis of nitrogen metabolism of different *S. cerevisiae* strains. The nitrogen uptake of the A9, M23, V80 and S288c strains was measured using the phenotype microarray technique. (**A**) Cluster analysis (Pearson coefficient, UPGMA) for similarity regrouping of tested strains on all nitrogen sources. I_N_ was used as a parameter. Values at the nodes represent cophenic correlation coefficients. (**B**) Each square represents the growth of one strain in the PM wells supplied with the indicated L-histidine containing dipeptide, as a nitrogen source. The extent of growth was generated from the tetrazolium dye reduction during 96 h and represented by the intensity of coloration; white squares mean no growth and dark black squares mean abundant growth. Dipeptides are grouped respect to the N-terminus amino acid.

According to the PM technique, the electron flow that results from the catabolism of nutritional substrates induces a shift in the tetrazolium dye to a purple color. When catabolism occurs at a subnormal rate, this results in a decrease in the electron flow and decreased intensity of the purple color [31]. On this basis, PM plates were used to test the ability of the strains to catabolize 380 different nitrogen sources, photographed every 15 min, to generate a growth curve for each well that primarily reflected dye reduction [25]. After 96 h of static incubation, PM analysis showed that the four strains differed greatly in the use of the nitrogen sources. In particular, while A9 and M23 catabolized 128 and 121 nitrogen sources, respectively, this number dramatically decreased for V80 and S288c, which used just 14 and 40 nitrogen sources, respectively (Fig. S1). Differences were observed also for the catabolism rate of nitrogen sources among strains. On the basis of cluster analysis of PM results, the four strains were ascribed to two groups, one consisting of A9 and M23, and the other one containing V80 and S288c (Fig. 1A). A9 and M23, but not V80 and S288c, grew slightly on different nucleotides, such as cytosine and adenine. They metabolized single L-amino acids, such as L-arginine, L-glutamine, L-phenylalanine, L-serine and L-tryptophan and showed high metabolic rates when fed with dipeptides containing alanine, valine, serine and threonine on their N-terminus. In parallel, all of the strains showed clear inability to metabolize dipeptides containing proline, asparagine, cysteine and lysine at their N-terminus. Notably, none of the strains grew in the L-histidine wells (Fig. S1). At the same time, A9 and M23 clearly did not grow in the presence of dipeptides containing L-histidine at their C- and/or N-terminus. On the contrary, strains V80 and S288c showed high and specific metabolic rates toward these dipeptides (Fig. 1B).

### L-histidine affects growth of *S. cerevisiae* flor yeasts

**Figure 2:**
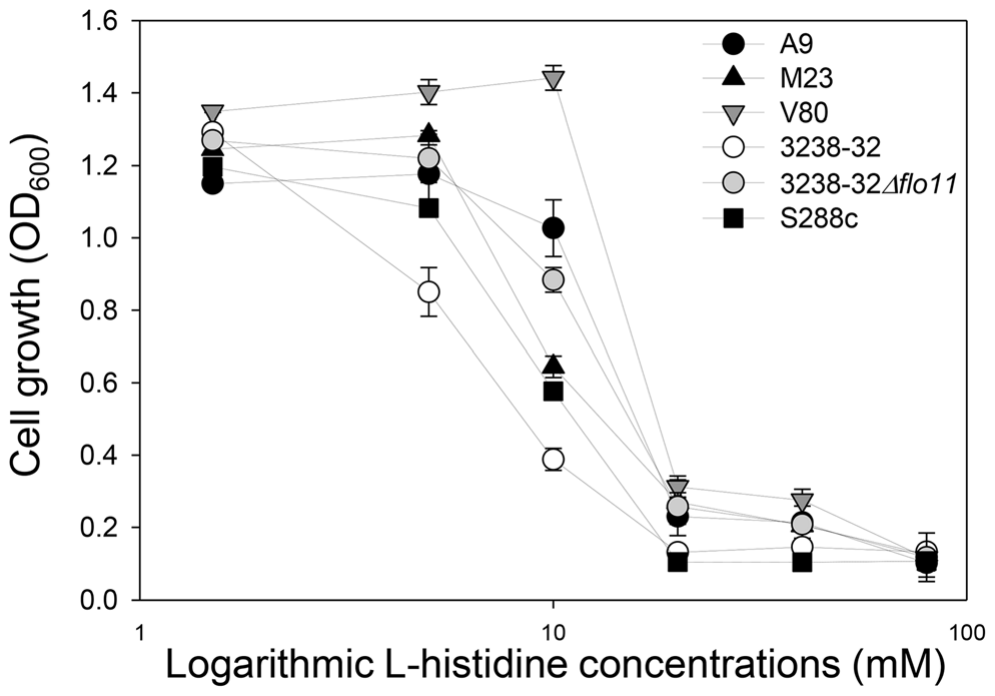
L-histidine affects the growth of different *S. cerevisiae* strains in YPD rich medium. Tested strains (10^4^ cells/mL) were incubated statically in 20% YPD for 48 h at 30°C, without (Ctrl) or with a serial dilution of L-histidine with concentrations range from 1 to 80 mM,. Dose-response curves show mean OD_600_ ± SD after 48 h of inoculation. *S. cerevisiae* flor strains are A9 (black circles), M23 (black triangles), V80 (grey down-pointing triangles), 3238-38 (white circles), 3238-32Δ*flo11* (grey circles) and S288c (black squares).

To further evaluate the inhibitory effects of L-histidine on *S. cerevisiae*, dye-independent growth measurements were carried out in 20% YPD medium added with up to 80 mM L-histidine. The OD_600_ of cell suspensions was measured after 48 h treatment with increasing concentrations of this amino acid. The L-histidine minimal inhibitory concentrations (MICs) ranged from 20 mM to 25 mM, and the half maximal inhibitory concentrations (IC_50_) were from 10 mM to 15 mM (Fig. 2). The diploid A9, M23 and V80 strains were slightly more resistant to higher L-histidine concentrations, with respect to the S288c haploid strain. Moreover, the four strains differed markedly in their tolerance to L-histidine. In the presence of 2.5 mM and 5.0 mM L-histidine, all of them increased the duration of the lag phase (Table 2). However, at these concentrations of L-histidine, strains V80 and S288c showed greater tolerance with respect to strains A9 and M23. Accordingly, the specific growth rate (μ) was not affected or was increased in strains V80 and S288c, while it was dramatically decreased in strains A9 and M23 (Table 2).

**Table 2:** Strains growth rate inhibition and Lag phase delay in the presence of different L-histidine concentrations.

	L-histidine [mM]							
	2.5		5		10		20	
Strain	μ inhibition (%)	Lag phase delay (h)	μ inhibition (%)	Lag phase delay (h)	μ inhibition (%)	Lag phase delay (h)	μ inhibition (%)	Lag phase delay (h)
**A9**	6.41	0.97	9.22 (a)	1.4	26.54 (b)	2.41	69.84	16.36
**M23**	8.4 (a)	1.65	25.09 (b)	1.68	30.92	5.58	63.27	8.37
**V80**	(−1.5) (c)	1.38	(−0.96) (c)	2.84	15.86	2.84	72.14 (d)	14.68
**3238-32**	(−2.01)	6.68	37.53	6.68	73.87 (d)	18.29	100 (e)	-
**3238-32Δ** ***flo11***	(−15.31)	3.79	(−8.5)	12.17	70.75	17.94	100 (e)	-
**S288c**	(−25.89)	0.66	(−5.31)	2.86	(−3.79)	9.47	98.35 (e)	-

The average of Log-phase specific growth rate was calculated by the DMFit software [26], and specific growth rate inhibition (%) and Lag-phase delay (h) of L-histidine treated cells were calculated in respect to control cells. Negative values in parentheses represent results with no growth rate inhibition. Values with the same letter are not statistically different (Multiple comparison analysis; 95% confidence). Minus symbol (-) represent a complete Lag-phase delay.

Previous studies have shown that V80 is characterized by low expression levels of Flo11p [21], while S288C does not express the *FLO11* gene due to a mutation in *FLO8* [24]. On the contrary, A9 and M23 showed high expression levels of the *FLO11* gene [21]. Thus, to evaluate possible correlations between tolerance to L-histidine and the expression levels of *FLO11*, the effects of L-histidine were evaluated also for the 3238-32 and 3238-32Δ*flo11* strains. 3238-32 is a haploid derivative of A9 that is characterized by high expression levels of *FLO11*, while 3238-32Δ*flo11* lacks Flo11p [3], [16]. Interestingly, these two strains showed dramatically different specific growth rate inhibition in the presence of L-histidine, and while 3238-32 behaved as A9 and M23, the behavior of 3238-32Δ*flo11* was comparable to that of V80 and S288C (Table 2).

L-histidine–containing dipeptides also had inhibitory effects on all of the tested strains, although at higher concentrations with respect to L-histidine (data not shown).

### L-histidine affects FLO11-associated phenotypes

**Figure 3:**
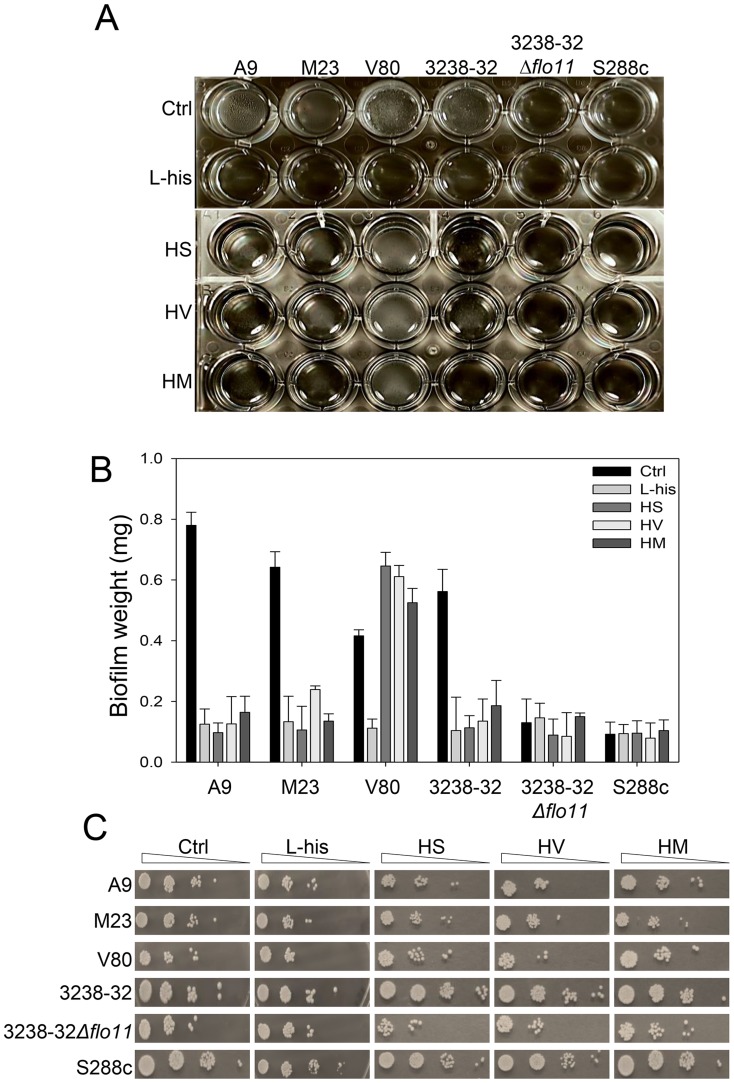
Biofilm formation of flor strains is inhibited by L-histidine. (**A**) Biofilm formation at the air-liquid interface in 24-well microtiter plates for strains A9, M23, V80, 3238-32, 3238-32Δ*flo11* and S288c after 5 days of static incubation in 1.5 mL flor medium at 30°C in the absence (Ctrl) and presence of 10 mM of L-histidine (L-his) and L-histidine–containing dipeptides. The biofilm is visualized as opaque floating material at the top of each well. (**B**) Dry weight determinations of the biofilms formed by the strains in (A) without (Ctrl) and with treatment with 10 mM L-histidine and the L-histidine–containing dipeptides (as indicated). Data are means +SD of three replicate treatments. (**C**) CFU recovery after plating on YPD agar using serial dilutions of a duplicate of all the strains/L-histidine and strains/dipeptides combinations.

To further investigate possible interactions between L-histidine and Flo11p, the effects of L-histidine were tested for biofilm forming ability and adherence to polystyrene for all of the strains in the flor medium. After 5 days of incubation in the presence of 10 mM L-histidine, strains A9, M23, V80, and 3238-32 showed dramatic reductions in air-liquid biofilm formation (Fig. 3A and 3B). This phenomenon was accompanied by minor reductions in the cell viabilities of these strains (Fig. 3C). S288c and 3238-32Δ*flo11* did not form an air–liquid biofilm, due to the lack of Flo11p, and their viability was not significantly affected by 10 mM L-histidine, as shown by the CFU recovery (Fig. 3C). Also, the addition of 10 mM dipeptides resulted in variations in biofilm formation after 5 days. Strains A9, M23 and 3238-32 did not form biofilms in the presence of all of the three dipeptides, and showed only a small reduction in CFU (Fig. 3), which was similar to that observed in the presence of L-histidine. On the contrary, V80 increased biofilm weight and viability in the presence of the dipeptides HM, HV and HS (Fig. 3).

**Figure 4:**
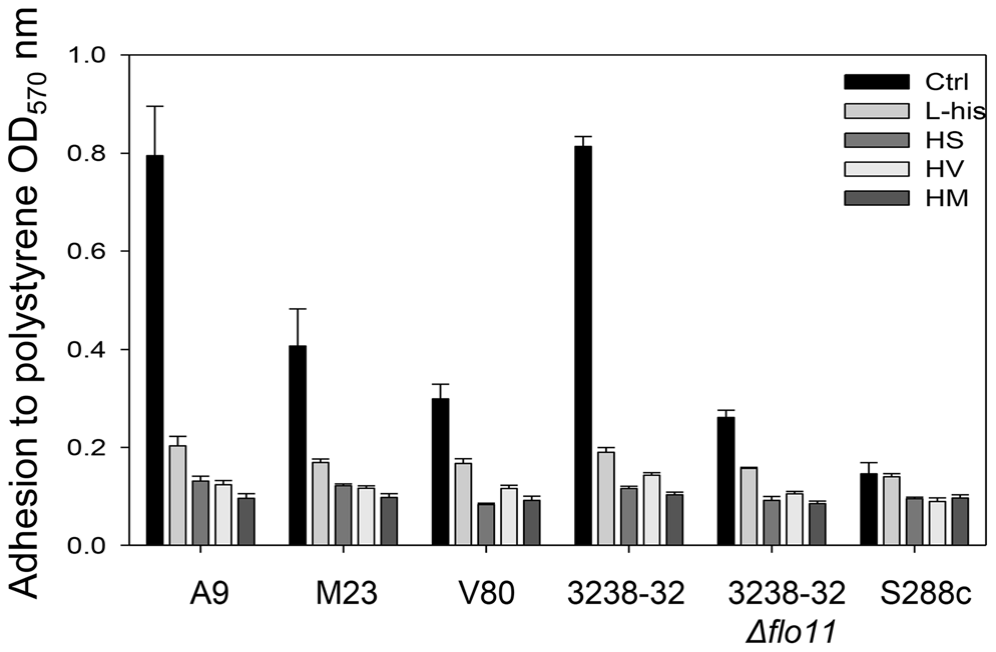
Loss of adhesion in presence of L-histidine and other dipeptides. Adhesion is expressed as OD_570_ and was measured using crystal violet dye after 48 h incubation of 5×10^6^ cell/mL of the *S. cerevisiae* strains in flor medium without (Ctrl) and with 10 mM L-histidine or the L-histidine–containing peptides.

Adhesion to polystyrene was evaluated after 48 h incubation in flor medium without or with 10 mM L-histidine or 10 mM dipeptides. S288c and 3238-32Δ*flo11* showed very low adhesion after 48 h, as expected for strains lacking Flo11p. However, strains A9, M23, V80, and 3238-32, which were highly adhesive in the absence of L-histidine, showed drastic reductions in their adhesion to polystyrene in the presence of L-histidine and the three dipeptides HV, HM and HS (Fig. 4).

Despite the above noted effect of L-histidine in inhibiting the *FLO11* associated phenotypes, the transcription analysis of *FLO11* in strain A9 (used as representative flor yeast strain) revealed that the addition of 10 mM L-histidine did not significantly (P = 0.763) affect *FLO11* transcription levels in flor medium.

### L-histidine induces modifications to the cell wall

**Figure 5:**
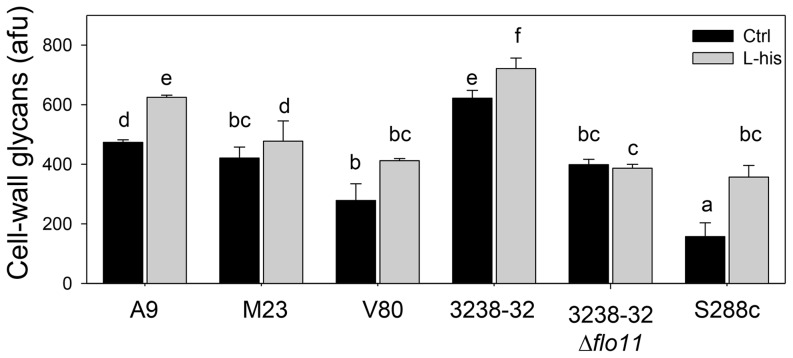
Modulation of cell-wall glycans of *S. cerevisiae* strains in the absence and presence of L-histidine. Cell-wall glycan levels (as arbitrary fluorescent units) without (Ctrl) and with 10 mM L-histidine treatment for A9, M23, V80, 3238-32, 3238-32Δ*flo11* and S288c strains (5×10^6^ cell/mL) in flor medium after 2 h. Data are means +SD from three replicate samples, of the fluorescence intensity of ConA-FITC bound to cell-wall glycans of 20.000 cells/sample. Multiple comparison analysis was conducted. Bars with the same letters are no statistically different (95% confidence).

To further investigate the inhibitory effects of L-histidine on biofilm formation, the fluorescence of concanavalin A–FITC-treated cells was analyzed using flow cytometry. This approach detects the levels of cell-wall protein mannosylation, which is a crucial factor in the biofilm formation process [32]. All of the strains showed enhancement in concanavalin A binding upon treatment with 10 mM L-histidine. The fluorescence intensity emitted by cells of A9, M23, V80, 3238-32, 3238-32Δ*flo11* and S288c varied (measured as arbitrary fluorescent units; afu), as shown in Figure 5. As the variation in cell fluorescence intensity directly reflects the variations in the contents of cell-wall glycans, which are mainly mannose residues, an enhancement of fluorescence in L-histidine–treated cells indicates an increase in cell-wall protein mannosylation.

**Figure 6:**
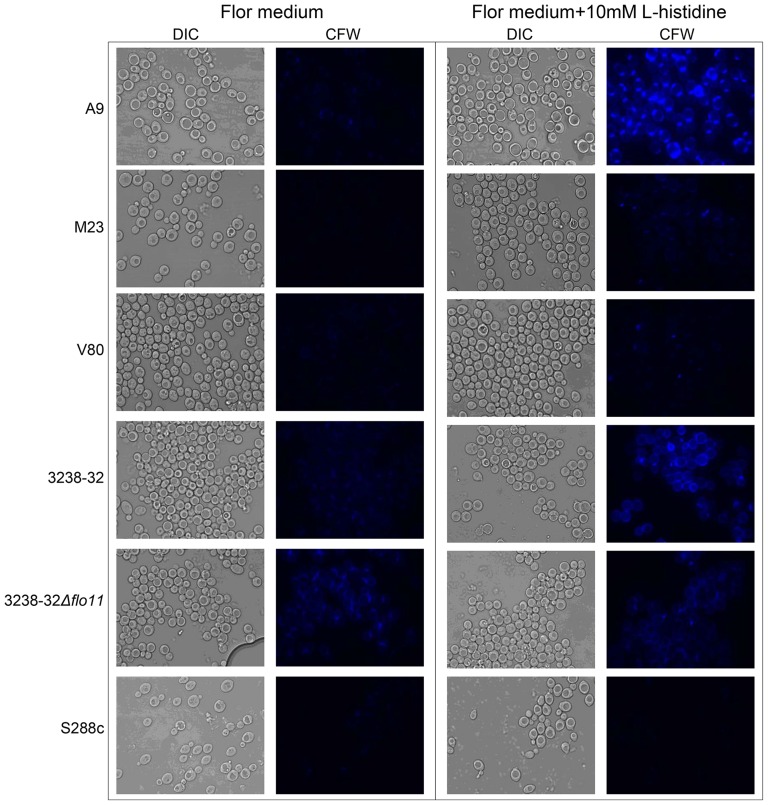
CFW staining of *S. cerevisiae* strains in the absence and presence of L-histidine. The A9, M23, V80, 3238-32, 3238-32Δ*flo11* and S288c strains (5×10^6^ cell/mL) were incubated for 2 h in flor medium without or with 10 mM L-histidine. After incubation, the samples were stained with 25 µM CFW for 5 min and observed by fluorescence microscopy. Bright-field differential interference contrast (DIC) and CFW images of the same field are shown. All of the images were captured under the same acquisition parameters and therefore reflect actual differences in CFW staining.

To determine whether these changes in mannosylation were accompanied by general cell-wall modifications induced by L-histidine, variations in the chitin content were also analyzed, according to Watanabe *et al.*, (2005) [33]. Fluorescence microscopy of CFW-stained cells showed remarkable differences in staining intensity among the strains that depended on the presence of L-histidine. In the absence of L-histidine, A9, M23, V80, 3238-32, and S288c showed low chitin content (Fig. 6). However, strains A9, M23 and 3238-32 increased chitin content upon L-histidine treatment, while V80, 3238-32Δ*flo11* and S288c did not show any significant variations. At the same time, CFW staining of the 3238-32Δ*flo11* strain was comparable both in the absence and presence of L-histidine. This might be due to constitutive over-production of chitin in cell wall related mutants, which will affect cell-wall integrity [34]–[35]

### FLO11 - L-histidine interaction model

**Figure 7:**
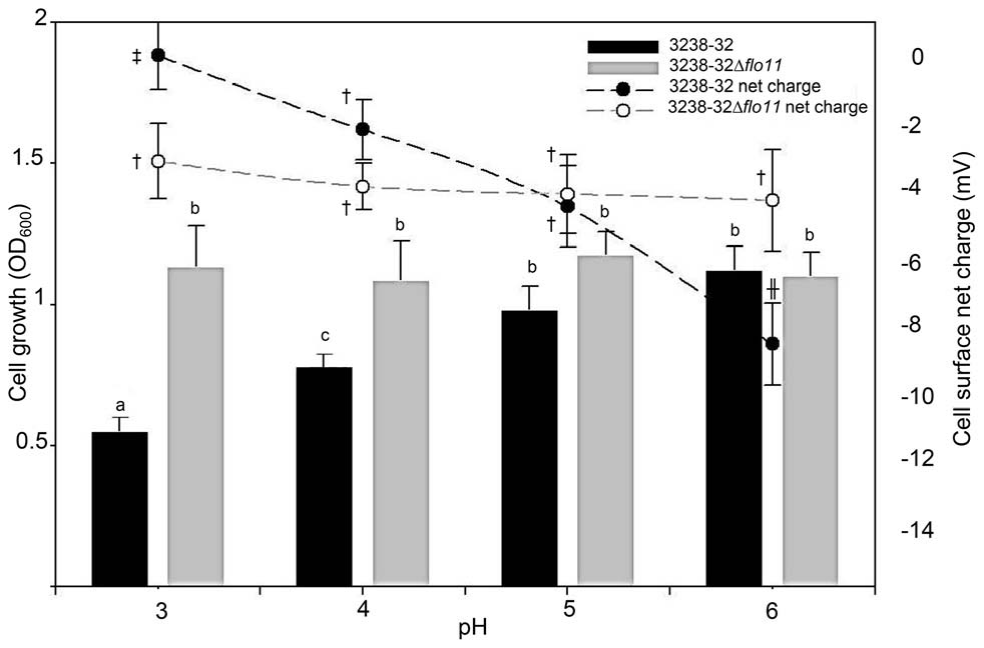
L-histidine effect on cell growth is *FLO11*-dependent, and related to pH and cell-surface charge. The histogram and the line graph illustrate the cell growth and the cell surface net charge, respectively, of the 3238-32 and 3238Δ*flo11* strains. Cells (10^4^ cells/mL) were incubated in SC minimal medium. The media were buffered at pH 3, 4, 5 and 6 using 0.1 M citric acid monohydrate and 0.2 M sodium phosphate. Cells were grown in 96-well microplates, statically at 30°C for 48 h. Growth was monitored measuring the OD_600_ in a SPECTROstar nano-microplate spectrophotometer (BMG Labtech, Germany). Cell surface net charge (Z-potential) was measured under the same conditions using the Zetasizer Ver. 6.20 (Malvern Instruments Ltd) after 48 h of treatment. Data are means ±SD of three replicates. A one-way analysis of varience (ANOVA) was performed and followed by Tukey honestly significant difference test (P<0.05). The analyses were performed independently for growth and cell surface charge data and values with the same letter/symbol are not statistically different.

To determine whether the effect of L-histidine is *FLO11*-dependent and/or mediated by physico-chemical interactions, strains 3238-32 and 3238-32Δ*flo11* were grown in SC medium buffered at pHs from 3.0 to 6.0. The 3238-32 growth performance changed depending on pH, and the cell density increased significantly with an increase in pH, reaching a maximum at pH 6.0. On the contrary, growth of 3238-32Δ*flo11* remained stable and independent of pH. At pH 6.0, the 3238-32 and 3238-32Δ*flo11* strains showed comparable growth (Fig. 7).

The cell surface net charge of 3238-32 also varied significantly, whereby at pH 3.0, it was slightly positive (+0.1±0.062 mV), while it decreased at increasing pH, to reach −8.5±0.087 mV at pH 6.0. In contrast, the cell surface net charge of 3238-32Δ*flo11* was stable, varying from −3.04±0.142 mV at pH 3.0 to −4.22±0.081 mV at pH 6.0. Interestingly, the increase in the anionic charge correlated with the increase in growth with the 3238-32 strain (Fig. 7).

Accordingly, TMR-HH stained cells of strains 3238-32 and 3238-32Δ*flo11* varied with pH. At pH 3.0, strains 3238-32 and 3238-32Δ*flo11* had low and comparable fluorescence, while at pH 6.0, the fluorescence intensity was notably enhanced in 3238-32, but not in 3238-32Δ*flo11* (Fig. S2).

## Discussion

Flor strains of *S. cerevisiae* yeast have the unique ability to form biofilms at the air–liquid interface of wine at the end of fermentation, when the medium is depleted of nutrients and further growth becomes dependent on oxygen. This multicellular growth is directly correlated with a series of rearrangements to the cell wall, in terms of the hydrophobicity and adhesion [21]. Indeed, *S. cerevisiae* can use either anaerobic or aerobic modes of substrate metabolism, which can induce specific changes to the cell at the level of the cell-wall organization, nutrient consumption, and cellular interactions with the surrounding environment [36]–[37].

Nitrogen is a fundamental nutrient in living cells, and its metabolism is involved in major developmental decisions in *S. cerevisiae* [38]. In nitrogen-starvation environment, some signaling pathways (i.e. TORC1, SPS-sensor and GAAC) that are largely related to nitrogen and amino-acid sensing and regulation have been shown to be involved in *FLO11* gene expression and multicellular growth in *S. cerevisiae* [39].

A previous study reported that clinical and vineyard isolates of *S. cerevisiae* can grow on a wide range of nitrogen sources, with respect to laboratory strains [40]. Accordingly, PM analysis in the present study showed similar behavior of the A9 and M23 flor strains, which metabolized more nitrogen sources with respect to the S288c laboratory strain. This reflects the high adaptation ability of these strains. However, this was not the case for the V80 strain, which showed a different behavior, similar to the laboratory strain S288c. Indeed, while V80 and S288c clearly metabolized L-histidine-containing dipeptides, A9 and M23 were definitely unable to grow on these dipeptides.

Dose-response analysis in nutrient-rich (dye-independent) medium showed that L-histidine not only does not support cellular growth as a nitrogen source, but its presence (concentrations ≥10 mM) reduces the growth rate, delays the lag-phase, and finally inhibits the growth of the tested strains. These effects were also observed in strains treated with higher concentrations of L-histidine–containing dipeptides. Other authors reported that L-carnosine, a L-histidine–containing dipeptide with potential antineoplastic effects [41], is able to slow cell growth rates and increase death of yeast cells in fermentative metabolism [37]. Interestingly, according to Letzien et al., (2014) [41], L-histidine mimicks the effect of L-carnosine although showing a stronger effect, similar to that observed in the present work.

In nutrient-depleted media, *S. cerevisiae* can trigger a series of stress-signaling pathways and responses, which include modulation of the cell wall, expression of the *FLO11* gene, and formation of biofilms [15], [18], [27]. This phenomenon was also observed in this study in the control wells of the biofilm-forming strains A9, M23, V80 and 3238-32, but not in the wells that contained L-histidine. In fact, the presence of 10 mM L-histidine was sufficient to completely inhibit biofilm formation and adhesion to polystyrene for all of the tested strains, and these major inhibitory effects were accompanied by minor reductions in cell viability. These inhibitory effects did not correlate with the transcription level of *FLO11*, which remained stable in the absence or the presence of L-histidine. The stability of *FLO11* expression levels evidences that L-histidine cannot be used as a nitrogen source, because if so, it would have been sensed by the GAAC pathway and/or the SPS sensor, leading to a repression of *FLO11* [4]–[7].

As stated before, cellular adhesion and binding are likely to be influenced by nonspecific interactions, such hydrophobic and electrostatic interactions [12]–[14]. Among the 20 naturally-occurring amino acids, L-histidine is a cationic amino acid with a unique imidazole ring as a side chain. These particular physico-chemical features make it a good candidate for nonspecific interactions. These would mainly be stacking and hydrogen-bond interactions, which would provide L-histidine with high affinity for cationic metals, aromatic amino acids, and many other compounds [42]–[43]. These features of L-histidine might induce the loss of cell adhesion and biofilm formation of the flor strains, by providing nonspecific physical interactions with the embedded cell-wall components in general, and with the highly O-mannosylated cell-wall mannoprotein Flo11p in particular. This leads to the failure of air–liquid biofilm formation and cell adhesion.

Cell-wall glycans and chitin are mainly responsible for cell permeability, and they are related to the cell-wall integrity pathways for responses to adverse conditions [10], [44]. The enhancement of fluorescence intensity of these cell-wall compounds in L-histidine–treated cells reveals the antimicrobial effects of this amino acid and reduces the permeability of the cell, which favors nonspecific interactions with cell-wall mannoproteins.

The proposed interaction model of 3238-32 and its isogenic 3238-32Δ*flo11* with L-histidine, showed that the effect of this amino acid is *FLO11*-dependent, and related to pH and cell-surface charge. In more detail, at pH 3.0, the repulsive interactions between the high cationic charge of L-histidine and the slightly positive cell-surface charge of the 3238-32 strain resulted in a reduction of 3238-32 growth. In parallel, at pH 6.0, the attractive interactions between the low cationic charged and neutralized imidazole ring of L-histidine (at the side-chain isoelectric point) and the high anionic cell surface charge of the 3238-32 strain led to the decreasing of the antimicrobial effects of this amino acid and thus an increasing of 3238-32 growth. These results highlight the role of *FLO11*, as 3238-32Δ*flo11* did not change its cell-surface charge and its interactions with L-histidine, and its growth. Similar behavior was seen by microscopic observations for these strains and TMR-HH: at pH 3.0, the low fluorescence intensity emitted from 3238-32 cells reflects the low adsorption of this dipeptide, which then increases at pH 6.0. Here again, 3238-32Δ*flo11* showed stable fluorescence intensity, and thus a stable interaction with the dipeptide.

The molecular mechanisms of this novel role of L-histidine are still unknown. Many studies have shown similar modes of action of several small cationic peptide sequences, with antimicrobial effects toward different fungi species. This is seen for human histatins and histidine-rich glycoproteins, which are directly involved in the host response to invasive growth of *Candida albicans*, with their binding to the cell-wall glycoprotein Msb2p [45]. A similar anti-adhesive behavior was reported for filastatin against some *Candida spp.* [46]. In contrast, hydrophobic interactions with the high cationic and hydrophobic hexapeptide PAF26 served as a bridge between some *S. cerevisiae* flor strains, to enhance biofilm formation [47]. To our knowledge, no previous studies have reported this mode of action of L-histidine. Interestingly, a recent study described a novel role of some D-amino acids in the triggering of bacterial biofilm disassembly. These D-amino acids did not affect the growth rate of bacterial cultures, and their mode of action is associated to their incorporation into the peptide side chains of the cell-wall peptidoglycan [48].

In conclusion, the main result in the present study relate to biofilm formation and adhesion ability. These findings reveal a novel biological activity of L-histidine that might be of high biotechnological interest. These data also suggest that glycosylated mucin-like proteins at the fungal cell wall, such as Flo11p, might be interacting partners for this unique amino acid. Future work will aim to explore the significance of these interactions in relation to the antimicrobial mechanisms of L-histidine with other non-flor yeast and filamentous fungi, and to determine the importance of protein glycosylation in this mechanism.

## Supporting Information

Figure S1 **High throughput analysis of nitrogen metabolism of different** ***S. cerevisiae*** **strains.** The nitrogen uptake of the A9, M23, V80 and S288c strains was measured using the phenotype microarray technique. Growth on nitrogen sources groups is showed and each square represents the growth of one strain in the PM wells supplied with a nitrogen source. The extent of growth was generated from the tetrazolium dye reduction during 96 h and represented by the intensity of coloration; white squares mean no growth and dark black squares mean abundant growth.
(TIF)

Figure S2 **Fluorescence microscopy of** ***S. cerevisiae*** **strains 3238-32 and 3238-32Δ** ***flo11*** **exposed to TMR-HH.** Cells (5×10^6^ cells/ml) were incubated in minimal medium with 1 mM of TMR-HH at 30°C for 2 h and subsequently with 25 µM CFW at 20°C for 5 min. Representative DIC bright-field as well as CFW, TMR, and CFW/TMR-overlay fluorescence micrographs of the same field are shown, for the different strains, as indicated.
(TIF)

Table S1 **Oligonucleotide primers used in this study.**
(DOCX)
