# Correction: L-Histidine Inhibits Biofilm Formation and *FLO11*-Associated Phenotypes in *Saccharomyces cerevisiae* Flor Yeasts

**DOI:** 10.1371/journal.pone.0118167

**Published:** 2015-01-30

**Authors:** The PLOS ONE Staff

There are errors in the published article. In the Abstract FLO11p should be replaced by Flo11p, and ∆flo11 should be replaced by flo11 mutant. In the section entitled “Cell surface charge variation of 3238-32 and the flo11 mutant in minimal medium at 222 different pHs” 3238∆flo11 should be replaced by 3238-32∆flo11.
