# Supplementary figures and images for: L-Histidine Inhibits Biofilm Formation and FLO11-Associated Phenotypes in Saccharomyces cerevisiae Flor Yeasts
Source: PLoS One. 2014 Nov 4;9(11):e112141. doi: 10.1371/journal.pone.0112141 (PMC4219837; doi:10.1371/journal.pone.0112141)

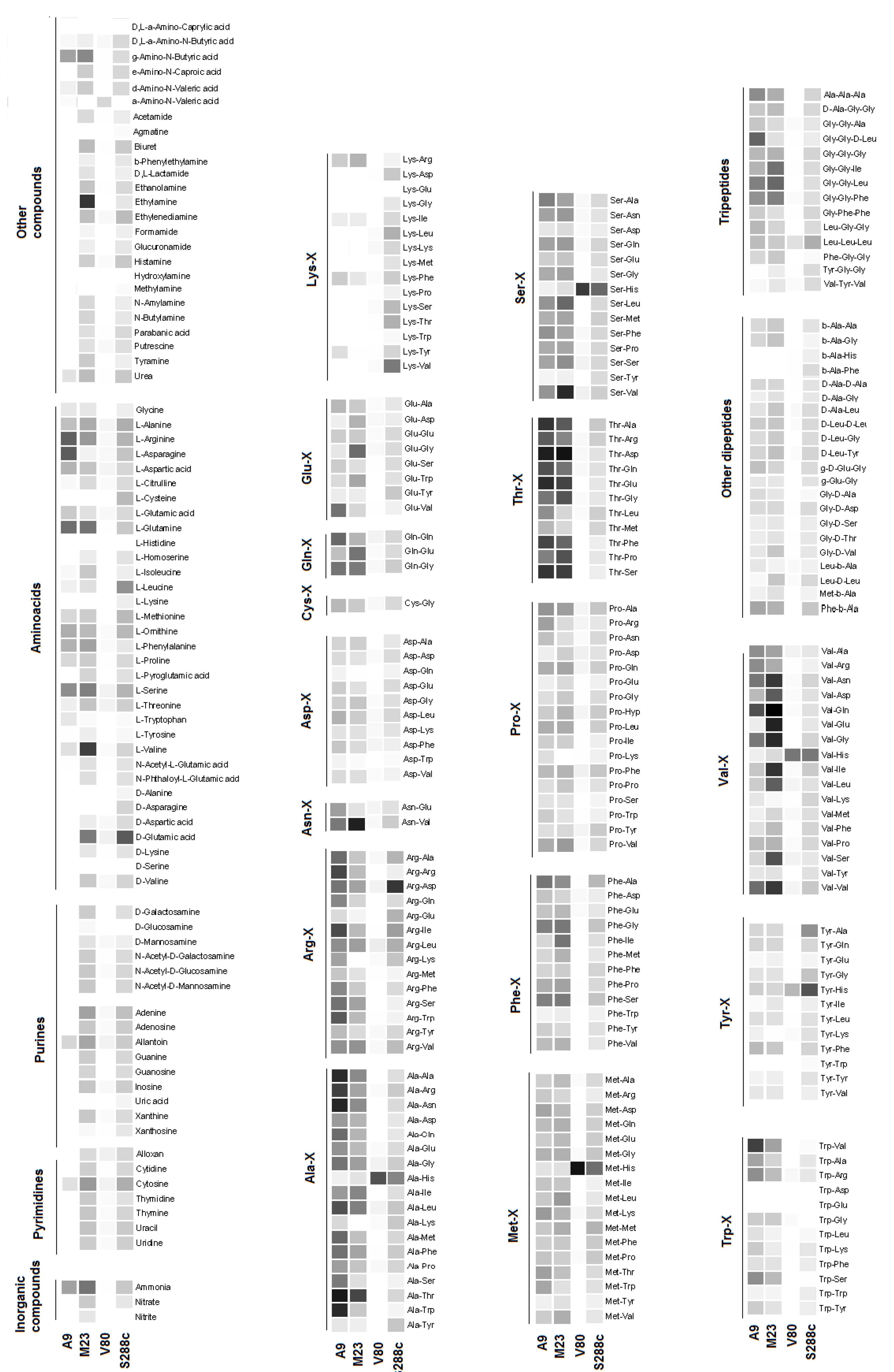

Supplement: Figure S1 — High throughput analysis of nitrogen metabolism of different S. cerevisiae strains. The nitrogen uptake of the A9, M23, V80 and S288c strains was measured using the phenotype microarray technique. Growth on nitrogen sources groups is showed and each square represents the growth of one strain in the PM wells supplied with a nitrogen source. The extent of growth was generated from the tetrazolium dye reduction during 96 h and represented by the intensity of coloration; white squares mean no growth and dark black squares mean abundant growth. (TIF) [file pone.0112141.s001.tif]

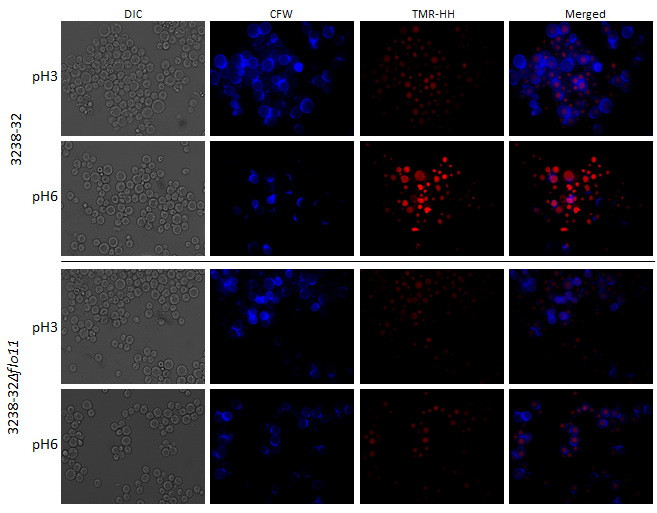

Supplement: Figure S2 — Fluorescence microscopy of S. cerevisiae strains 3238-32 and 3238-32Δ flo11 exposed to TMR-HH. Cells (5×10^6^ cells/ml) were incubated in minimal medium with 1 mM of TMR-HH at 30°C for 2 h and subsequently with 25 µM CFW at 20°C for 5 min. Representative DIC bright-field as well as CFW, TMR, and CFW/TMR-overlay fluorescence micrographs of the same field are shown, for the different strains, as indicated. (TIF) [file pone.0112141.s002.tif]
