# Supplementary material for: L-Histidine Inhibits Biofilm Formation and FLO11-Associated Phenotypes in Saccharomyces cerevisiae Flor Yeasts
Source: PLoS One. 2014 Nov 4;9(11):e112141. doi: 10.1371/journal.pone.0112141 (PMC4219837; doi:10.1371/journal.pone.0112141)
Supplement: Table S1 — Oligonucleotide primers used in this study. (DOCX) [file pone.0112141.s003.docx]

| **Gene** | **Primer** | **Sequence 5´- 3´** |
| --- | --- | --- |
| *FLO11* | Forward | 5’-AGGTTCAAATGGTGCCAAGA-3’ |
| *FLO11* | Reverse | 5’-AGCCACGCTAGAAGCAGAAG-3’ |
| *ALG9* | Forward | 5´-CACGGATAGTGGCTTTGGTG-3´ |
| *ALG9* | Reverse | 5´-GGCAGCAGGAAAGAACTTGGG-3´ |
| *TAF10* | Forward | 5´-CAGGATCAGGTCTTCCGTAGC-3´ |
| *TAF10* | Reverse | 5´-GTAGTCTTCTCATTCTGTTGATG-3´ |
| *UBC6* | Forward | 5´-GATACTTGGAATCCTGGCTGG-3´ |
| *UBC6* | Reverse | 5´-GGGTCTTCTGTTTCATCACCTG-3´ |
